# Variable Patterning of Chromatin Remodeling, Telomere Positioning, Synapsis, and Chiasma Formation of Individual Rye Chromosomes in Meiosis of Wheat-Rye Additions

**DOI:** 10.3389/fpls.2018.00880

**Published:** 2018-07-02

**Authors:** Tomás Naranjo

**Affiliations:** Departamento de Genética, Fisiología y Microbiología, Facultad de Biología, Universidad Complutense de Madrid, Madrid, Spain

**Keywords:** chromatin remodeling, telomere dynamics, synapsis, chiasmata, rye, wheat, FISH

## Abstract

Meiosis, the type of cell division that halves the chromosome number, shows a considerable degree of diversity among species. Unraveling molecular mechanisms of the meiotic machinery has been mainly based on meiotic mutants, where the effects of a change were assessed on chromosomes of the particular species. An alternative approach is to study the meiotic behavior of the chromosomes introgressed into different genetic backgrounds. As an allohexaploid, common wheat tolerates introgression of chromosomes from related species, such as rye. The behavior of individual pairs of rye homologues added to wheat has been monitored in meiotic prophase I and metaphase I. Chromosome 4R increased its length in early prophase I much more than other chromosomes studied, implying chromosome specific patterns of chromatin organization. Chromosome conformation affected clustering of telomeres but not their dispersion. Telomeres of the short arm of submetacentric chromosomes 4R, 5R, and 6R failed more often to be included in the telomere cluster either than the telomeres of the long arms or telomeres of metacentrics such as 2R, 3R, and 7R. The disturbed migration of the telomeres of 5RS and 6RS was associated with failure of synapsis and chiasma formation. However, despite the failed convergence of its telomere, the 4RS arm developed normal synapsis, perhaps because the strong increase of its length in early prophase I facilitated homologous encounters in intercalary regions. Surprisingly, chiasma frequencies in both arms of 4R were reduced. Similarly, the short arm of metacentric chromosome 2R often failed to form chiasmata despite normal synapsis. Chromosomes 1R, 3R, and 7R showed a regular meiotic behavior. These observations are discussed in the context of the behavior that these chromosomes show in rye itself.

## Introduction

Bivalents formed during meiotic prophase I are essential to halve the chromosome number. Bivalent formation requires that homologous chromosomes, mainly located in separate nuclear territories in premeiotic interphase nuclei (Bass et al., 2000; Maestra et al., 2002), move to find one another. In many species, concomitant with a chromatin remodeling process that causes a considerable chromosome elongation during leptotene, telomeres undergo an oriented migration and converge in a tight cluster in a small area of the nuclear envelop. This suprachromosomal meiotic configuration, the so-called bouquet, facilitates chromosome interactions that culminate in the identification of the homologous partner (Bass et al., 1997, 2000; Niwa et al., 2000; Trelles-Sticken et al., 2000; Cowan et al., 2001; Scherthan, 2001). The bouquet is disorganized once homologues undergo synapsis.

A programed production of double-strand DNA breaks (DSBs) catalyzed by Spo11 in conjunction with other proteins triggers the initiation of chromosome interactions (Keeney et al., 1997; Neale and Keeney, 2006). DSBs are resected to generate 3′ single-strand DNA overhangs that bind recombinases RAD51 and DMC1. These nucleoprotein filaments invade a double-strand DNA stretch of the homologous chromosome to find its complementary strand (Hunter and Kleckner, 2001). During leptotene, chromosomes form the axial element, a protein-rich backbone that will keep the sister chromatids together until the second division. The search of a repair template in the homologous chromosome is instigated by proteins of the axis such as Hop1-Red1 in budding yeast, ASY1-ASY3 in *Arabidopsis* and PAIR2-PAIR3 in rice (Hollingsworth and Byers, 1989; Thompson and Roeder, 1989; Caryl et al., 2000; Nonomura et al., 2006; Wang et al., 2011; Ferdous et al., 2012). The identification of the homologous partner in the invaded chromatid is necessary for chromosome pairing and synapsis in many organisms (Roeder, 1997; Baudat et al., 2000; Romanienko and Camerini-Otero, 2000). Then, homologues become aligned, form the tripartite synaptonemal complex (SC) during zygotene, and are fully synapsed at pachytene (Page and Hawley, 2004). The SC maintains homologues in close juxtaposition along their length and serves as a scaffold for factors of the recombinational repairing machinery (Zickler and Kleckner, 2015). The genetic control of the repairing machinery ensures a minimum of one crossover (CO) per homologous pair. However, repair of the majority (95%) of DSBs produced in plants and animals culminates in a non-crossover (NCO) (Higgins et al., 2014). The SC disassembles at diplotene, once COs formation is completed, and chromatin undergoes a progressive condensation. Meanwhile, homologues remain physically connected by chiasmata, the cytological expression of COs, until their disjunction in anaphase I.

Deviations from this meiotic prophase I program have been reported in several organisms (reviewed in Zickler and Kleckner, 2016). In fission yeast, homologous pairing is recombination-independent and COs are formed in the absence of SC (Ding et al., 2004). In *Drosophila melanogaster* females and in *Caenorhabditis elegans*, pairing and synapsis can occur independently of recombination (Lake and Hawley, 2012; Rog and Dernburg, 2013). However, in both cases, DSBs are produced after SC formation. The *D. melanogaster* males and the silk worm (*Bombyx mori*) females both with achiasmate meiosis, show well-aligned bivalents at metaphase I. The presence of the SC until anaphase I provides the physical connection between homologues in *B. mori* females (Rasmussen, 1977). *B. mori* males form chiasmate bivalents. In *D. melanogaster* males, which do not form SC, pairing initiates at a specific site in sex chromosomes, and probably in chromosome 4, but no pairing center has been found in the other two autosomes (Tsai and McKee, 2011). Sex differences in the meiotic process have been observed in other organisms such as, planarian worms (Pastor and Callan, 1952; Oakley and Jones, 1982; Oakley, 1982), *Lilium* and *Fritillaria* (Fogwill, 1958), grasshoppers (Perry and Jones, 1974), *Arabidopsis thaliana* (Drouaud et al., 2007), mice (Petkov et al., 2007), or humans (Hou et al., 2013).

Studies aimed at unraveling the molecular mechanisms underlying the chromosome dynamics in meiotic prophase I are based on the use of meiotic mutants and often focus on the full chromosome complement, neglecting the behavior of individual chromosomes. However, individual chromosomes may respond in different way to the general program of the meiotic cell. An apparent example of this differential behavior is the case of the sex chromosomes in the heterogametic sex of species with XX/XY, XX/X0, or ZZ/ZW. Non-homologous regions of sex chromosomes appear usually unmatched and with apparent changes in the chromatin organization during prophase I, which, in the case of mammals, is accompanied by silencing of unsynapsed chromatin (Page et al., 2006; Turner, 2013). It is also remarkable the variable chromosome behavior in spermatocytes of the planarian worm *Mesostoma ehrenbergii ehrenbergii.* Chiasma formation is extremely restricted: three homologous pairs form one distal chiasmata and appear as bivalents at metaphase I, while the other two pairs do not form chiasmata and appear as univalents (Croft and Jones, 1989). Chromosomes of common wheat *Triticum aestivum* show differences in the chiasma distribution and in their ability to form chiasmata after deletion of terminal regions (Naranjo, 2015).

An alternative approach to the use of meiotic mutants in a given species is the study of the meiotic behavior of its chromosomes when they are introgressed in the genetic background of a different species. Common wheat (genome formula, AABBDD, 2n = 6x = 42) as an allohexaploid, tolerates the addition of chromosomes from related species such as rye, *Secale cereale* (RR, 2n = 14). A handful of complete sets of wheat-rye additions are available (Lukaszewski, 2015). These lines can be used in the study of the behavior of individual rye chromosomes both in somatic and meiotic cells, in the assignation of genetic markers to chromosomes, or in the study of genetic interactions between wheat and rye chromosomes. In addition they represent an excellent start point for the introgression of useful genes of rye into wheat.

Painting of the rye chromosomes present in each disomic addition allows to monitor their pairing and synapsis and to estimate the number of chiasmata formed (Maestra et al., 2002; Corredor and Naranjo, 2007; Naranjo and Corredor, 2008; Naranjo et al., 2010; Valenzuela et al., 2012; Naranjo, 2014, 2015). Rye chromosomes bear apparent subtelomeric heterochromatin blocks (Darvey and Gustafson, 1975), which can be visualized by fluorescence *in situ* hybridization (FISH) making possible to identify the position of the adjacent telomere in the meiotic bouquet (Naranjo et al., 2010; Naranjo, 2014). The position of the telomere of the short arm of chromosome 5R (5RS) at the bouquet depends of the length of the other arm of this submetacentric chromosome. The 5RS arm of the standard chromosome 5R is much shorter than the long arm (5RL), but in a truncated chromosome 5R (del5R) lacking the last 70% distal of 5RL (del5RL) both arms have a similar length. Although the 5RS arm is the same in chromosomes 5R and del5R, its telomere fails more often in its incorporation to the telomere cluster in the standard chromosome than in the truncated one. Disturbed telomere migration has a negative effect on the development of synapsis and chiasma formation (Naranjo et al., 2010). Chromosomes 1R and 6R carry subtelomeric heterochromatin chromomeres in both ends. This allowed to verify that the end of their long arms, 1RL and 6RL, were included in the telomere cluster in almost all cells (>99%) while one or both telomeres of 1RS, or 6RS, were separated of the telomere cluster in 27 and 40% of meiocytes, respectively. Thus, incomplete telomere migration occurs more often in the short arm of the submetacentric chromosome 6R than in the short arm of the almost metacentric chromosome 1R. Failure of synapsis and chiasma formation is also relatively frequent in the 6RS arm (Naranjo, 2014). This result is consistent with the behavior of 5RS in chromosomes 5R and del5R.

The meiotic behavior of chromosomes 1R, 5R, and 6R in a wheat background suggests that the genetic and structural chromosome identity is manifested in the appearance of chromosome specific features in the development of the meiotic program. Verification of this idea implies the analysis of all individual chromosomes. In the case of rye, it remains to be studied the meiotic behavior of metacentric chromosomes 2R, 3R, and 7R and submetacentric chromosome, 4R. On the other hand, from the effect of chromosome structure on telomere clustering the question arises whether telomere dynamics during bouquet dissolution is also chromosome conformation-dependent or not. The aim of this paper is to carry out a comparative study of the dynamics of individual rye chromosomes in a wheat background during prophase I. Three different aspects of the meiotic process are concerned: (i) chromatin remodeling (ii) the telomere movement leading to the formation of the bouquet and its resolution; (iii) the effect of telomere positioning at the bouquet on synapsis and chiasma formation.

## Materials and Methods

### Plant Material

Seven wheat-rye introgression lines, each carrying one of the seven chromosome pairs of rye (*S. cereale*, 2n = 14) introgressed in hexaploid wheat *T. aestivum*, were used. The wheat-2R, wheat-3R, wheat-4R, wheat-5R, wheat-6R, and wheat-7R lines are disomic additions of the Imperial rye chromosome in a Chinese Spring wheat background (Driscoll and Sears, 1971). The wheat-1R line is a disomic substitution for chromosome 1A generated in hexaploid wheat Pavon 76 (Lukaszewski, 2008), and used for other purpose (Valenzuela et al., 2012). All wheat-rye introgression lines used were provided by A. J. Lukaszeski. The chromosome constitution of the plants studied was verified by FISH analysis of root tips in squashed preparations as described for meiosis. Seeds were germinated in November and grew in a greenhouse under natural light. Spikes at meiosis were cut and checked to establish the meiotic stage of each flower, One anther per flower was examined. When this anther was at prophase I or metaphase I the other 2 were fixed in 3:1 ethanol: acetic acid and stored at 4°C.

### Fluorescence *in Situ* Hybridization

Preparations of fixed anthers were carried out as described (Maestra et al., 2002). Telomeres of wheat and rye chromosomes were labeled with the pAtT4 DNA probe, from *Arabidopsis* (Richards and Ausubel, 1988). The arrangement of telomere was used to identify the leptotene, zygotene, and pachytene stages as described (Corredor et al., 2007; Naranjo et al., 2010). Subtelomeric chromomeres of rye were visualized using clone pSc74, which contains a rye-specific 480-bp tandem repeat (Bedbrook et al., 1980; Cuadrado and Schwarzacher, 1998). The subtelomeric pSc74 signal identified the position of the adjacent rye telomere. Rye centromeres labeled with the rye-specific clone pAWRC.1 (Franki, 2001) were positioned at the bouquet and in the bivalents at pachytene and metaphase I. A fourth DNA probe, pUCM600, containing a rye-specific repeat (Gonzalez-Garcia et al., 2011) was added to paint each rye bivalent and quantify its level of synapsis in Pollen Mother Cells (PMCs) at pachytene. The probes, pUCM600, pAWRC.1, and pSc74, were used for the identification of each rye bivalent and the arms associated at metaphase I. Concentrations of DNA probes in the hybridization mixes were as described (Naranjo et al., 2010) In the analysis of the position of rye telomeres at the bouquet, probes pAtT4 and pAWRC.1 were labeled with biotin-16-dUTP and probe pSc74 with digoxigenin-11-dUTP. For painting of the rye bivalent at pachytene and metaphase I, the rye-specific DNA probes pAWRC.1 and pUCM600 were labeled with biotin-11-dUTP, while either digoxigenin-11-dUTP or biotin-16-dUTP were used in the case of probe pSc74. In nuclei at pachytene, the telomeres of wheat and rye chromosomes were visualized with the pAtT4 DNA probe labeled with digoxigenin-11-dUTP. Labeled probes were detected as described (Naranjo et al., 2010).

### Fluorescence Microscopy and Image Processing

Cells at mitotic metaphases and PMCs at pachytene and metaphase I were viewed under an Olympus BX60 fluorescence microscope equipped with an Olympus DP70 CCD camera. Cells at the bouquet stage were studied under an Olympus BX61 fluorescence microscope and processed as described (Naranjo, 2014)

### Chromosome Length and Telomere Arrangement

The length of rye chromosomes in both mitotic metaphases and meiocytes at pachytene, as well as different distances in nuclei at the bouquet stage and pachytene, were measured using the Adobe Photoshop CS4 software. In each wheat-rye addition line, the lengths of 10 mitotic chromosomes and 10 completely synapsed pachytene bivalents were scored. Separation between the rye chromosome ends, and the major axis of each nucleus were measured in 2D projections of cells at the bouquet stage and pachytene. The distance of the ends of each rye chromosome to the center of the telomere cluster was also measured in cells at the bouquet. An average number of 100 cells per meiotic stage and line were scored.

## Results

### Rye Chromatin Remodeling During Leptotene Shows a Distinctive Pattern in the Wheat-4R Addition

**FIGURE 1:**
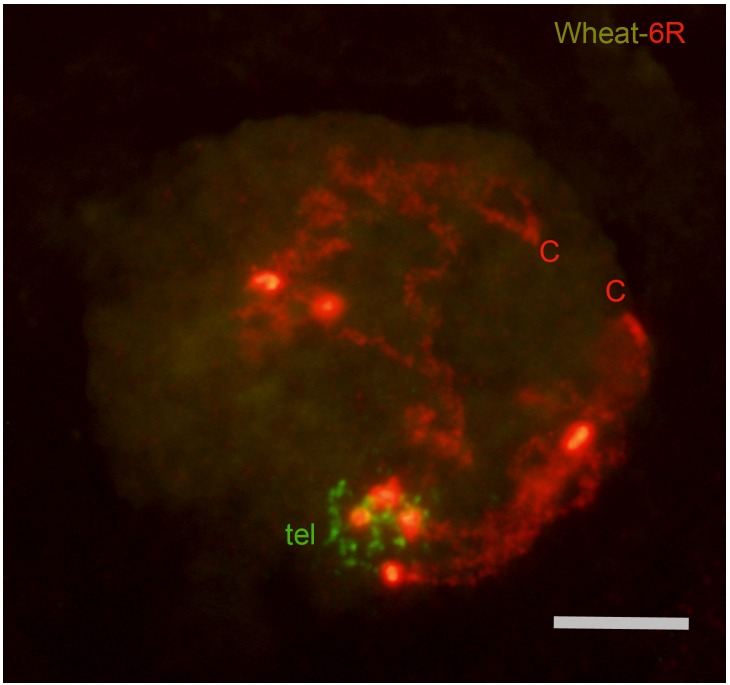
Nucleus of the wheat-6R addition at the bouquet stage. Chromatin folding makes it difficult to trace the chromosomal axis along its entire length. Centromeres (C) and telomeres (tel) are indicated. Bar represents 10 μm.

**FIGURE 2:**
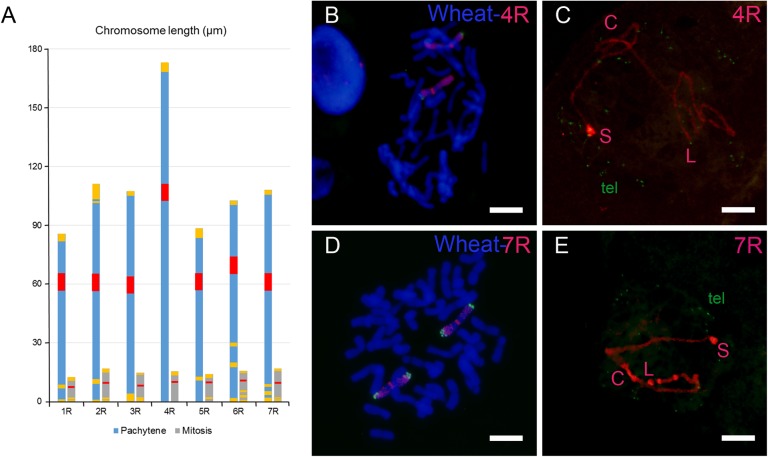
Chromosomes of rye in mitosis and pachytene in wheat-rye additions. **(A)** Karyotypes showing the different size of mitotic and meiotic chromosomes. The position of the centromere (red) and heterochromatic chromomeres (yellow) is indicated. **(B)** Mitotic metaphase of the wheat-4R addition. **(C)** Bivalent 4R at pachytene. Green signals correspond to telomeres (tel). **(D)** Mitotic metaphase of the wheat-7R addition. **(E)** Bivalent 7R at pachytene. Bars represent 10 μm.

Concomitant with the bouquet organization, chromatin undergoes a remodeling process that reduces its packaging level and causes a large elongation of chromosomes. At the bouquet, decondensation of rye chromatin was apparent but a considerable folding degree was still observed (**Figure 1**). This remnant folding of chromatin made it difficult to trace and measure the complete chromosome length. The assessment of chromatin reorganization in each rye chromosome was based on the variation of its length in completely synapsed bivalents at pachytene relative to that in mitotic metaphase. **Figure 2** compares the karyotype of rye chromosomes in both types of cells. In addition to the chromosome length, karyotypes show the position of the centromere and heterochromatin chromomeres. The two arms of all rye chromosomes show a distinctive pattern of heterochromatin markers. Chromosomes 4R and 5R bear a subtelomeric chromomere only in the short arm, the 4RL arm shows no marker and the subdistal chromomere of 5RL is smaller than that of 5RS. The subtelomeric chromomeres of the short and long arms of chromosomes 1R, 2R, 3R, 6R, and 7R differ in the heterochromatin contain. In addition to the subtelomeric marker, the arms 1RL, 2RS, 2RL, 6RL, and 7RL carry one or two intercalary chromomeres. Values of chromosome length and centromere index (100 × short arm length/chromosome length) are given in **Table 1**. The length of mitotic chromosomes ranges between 12.9 and 16.4 μm with an average of 15.1 μm. Bivalents at pachytene of chromosomes 1R, 2R, 3R, 5R, 6R, and 7R reached a length that ranges between six and seven times the size of the mitotic chromosome. Surprisingly, chromosome 4R is 11.2 times longer in pachytene than in mitotic metaphase. This result supports a different reorganization pattern of rye chromatin in leptotene in the wheat-4R addition relative to the others.

**Table 1:** Length (μm) of rye chromosomes in mitotic metaphase (M) and pachytene (P), P/M ratio and centromere index (100 × short arm length/chromosome length) in wheat-rye additions.

Chromosome	M	P	P/M ratio	Centromere
				index
1R	14.2 ± 2.1	85.6 ± 5.3	6.0	37
2R	16.4 ± 3.4	111.1 ± 5.7	6.8	46
3R	15.3 ± 1.2	107.3 ± 12.0	7.0	46
4R	15.4 ± 0.6	172.8 ± 23.2	11.2	39
5R	12.9 ± 1.3	88.5 ± 15.2	6.9	33
6R	15.8 ± 2.5	102.6 ± 7.7	6.5	33
7R	15.7 ± 1.7	108.2 ± 9.4	7.0	45


### Clustering, but Not Dispersion, of Telomeres Is Chromosome Conformation-Dependent

**FIGURE 3:**
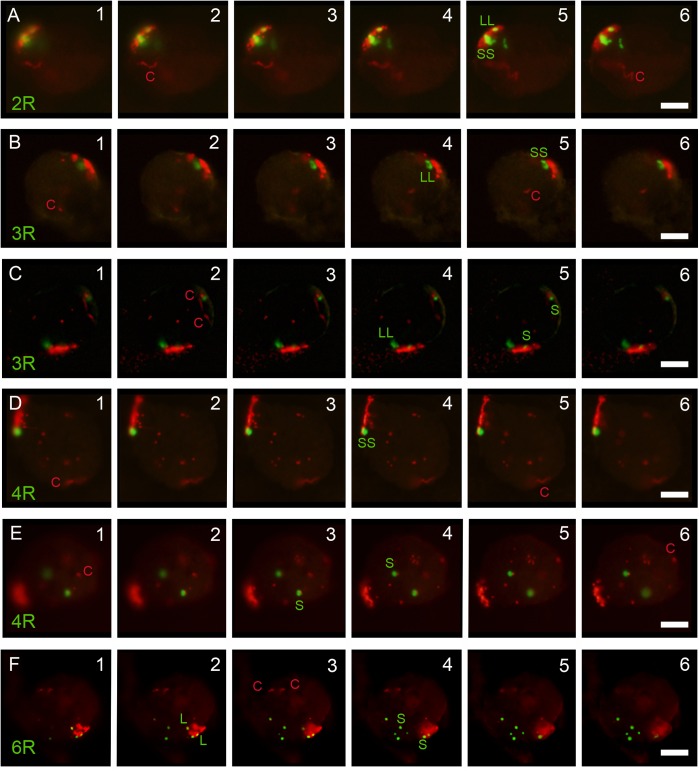
3D analysis of the arrangement of the subtelomeric chromomeres (green) of the short (S) and long (L) arm or rye chromosomes at the bouquet in wheat-rye additions. The telomere cluster (red) and the rye centromeres (c) are indicated. **(A)** Nucleus with the two ends of 2R at the telomere cluster. Homologous chromomeres of both arms are associated. **(B)** The subtelomeric chromomeres of 3RS and 3RL are associated and incorporated to the telomere cluster. **(C)** The subtelomeric chromomeres of 3RL are associated and incorporated to the telomere cluster. Only one 3RS telomere is in the telomere cluster. **(D)** The ends of 4RS are associated and locate at the telomere cluster. **(E)** The 4RS telomeres are separated and locate out of the telomere cluster. **(F)** Both ends of the chromosome pair 6R are separated, the two 6RL telomeres and one telomere of 6RS are included in the telomere cluster. Bars represent 10 μm.

**FIGURE 4:**
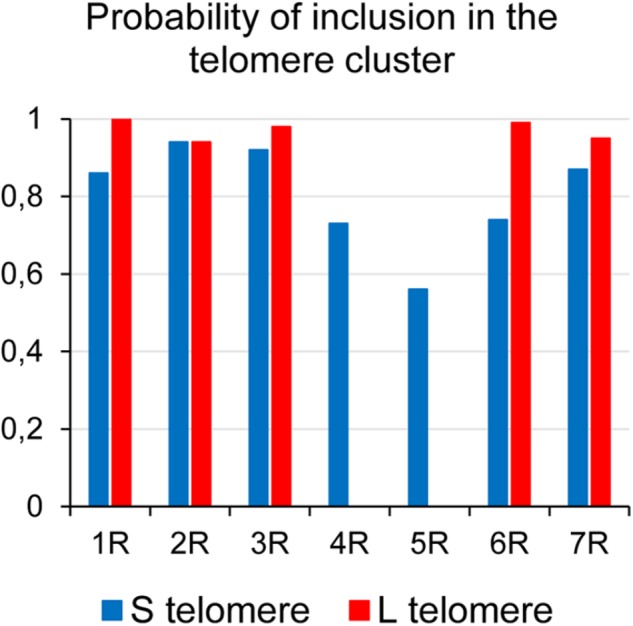
Probability of inclusion in the telomere cluster of the telomeres of rye chromosome arms carrying subtelomeric chromomeres in wheat-rye additions.

The position of the telomere of the short arm of all rye chromosomes and of the long arms 1RL, 2RL, 3RL, 6RL, and 7RL in PMCs at early prophase I was identified by its close proximity to the adjacent subtelomeric heterochromatin marker. FISH analysis combining the telomeric and the rye-specific heterochromatin DNA probes in nuclei at the bouquet stage revealed whether rye telomeres were included in the telomere cluster or not. **Figure 3** shows nuclei with the two ends of chromosomes 2R and 3R, and the end of chromosome arm 4RS at the telomere cluster (**Figures 3A,B,D**, respectively). In all of these cases the homologous chromosome ends are intimately associated denoting the initiation of synapsis. The telomere of one chromosome arm 3RS, one chromosome arm 6RS and the two arms 4RS are not included in the telomere cluster in nuclei of **Figures 3C,E,F**, respectively. From the number of cells with zero, one or two homologous ends included in the telomere pole it was possible to estimate the probability of incorporation to the telomere cluster of the telomere of the seven short chromosome arms and the long arms 1RL, 2RL, 3RL, 6RL, and 7RL. This probability appears diagrammed in **Figure 4**. The telomere of both arms of metacentric chromosomes 2R, 3R, and 7R as well as the telomere of 1RL and 6RL migrated to the telomere pole in almost all cells. However, the telomere of the short arm of submetacentric chromosomes 4R, 5R, and 6R failed in its incorporation to the telomere pole in more than 25% of the meiocytes. On average, such telomeres were separated 10 μm from the telomere cluster. Accordingly, the probability of incorporation of the telomere of the short arm of the seven rye chromosomes and the centromere index were positively correlated (*r* = 0.828, *t* = 13.16, d.f. = 5, and *p* < 0.01). This result supports an effect of chromosome conformation in the positioning of telomeres during the organization of the bouquet. Although the centromere index of 1R identifies this chromosome as submetacentric, the location of the nucleolar organizing region on 1RS probably represents an additional factor conditioning its arrangement in leptotene. Consistent with the disturbed positioning of the telomeres of 4RS, 5RS, and 6RS is the reduction in the frequency of association of their subtelomeric markers in cells with the two homologous ends included in the telomere cluster. While the subtelomeric chromomeres of 4RS, 5RS, and 6RS were intimately associated on average in 46% of the cells studied, the homologous ends of metacentric chromosomes were in 86%.

**FIGURE 5:**
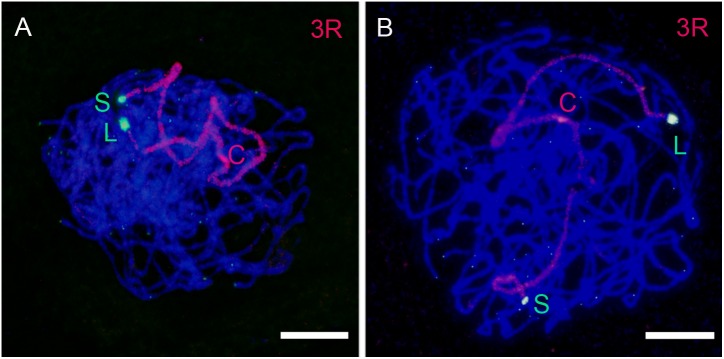
Variable separation of the ends of the rye bivalent in pachytene nuclei of the wheat-3R addition. **(A)** Chromosome ends closely located. **(B)** Very distant chromosome ends. Bars represent 10 μm.

The fact that telomere clustering was chromosome conformation dependent led to verify whether the dispersive telomere movement responsible of the bouquet disorganization was also affected by chromosome conformation or not. When both ends of rye chromosome pairs 1R, 2R, 3R, 6R, or 7R were included in the telomere cluster, they were situated at an average distance of 2 mμ. Such a distance is expected to increase because of telomere dispersion during zygotene. Thus, the spatial separation between the ends of each bivalent in nuclei at pachytene, in which the bouquet is completely disorganized, should provide an assessment of the effect of the dispersive telomere movement. The distance between the chromosome ends was scored in completely synapsed bivalents. A great between cells variation was found. **Figure 5** shows nuclei with the chromosomal ends of the rye bivalent relatively close or occupying diametrically opposed positions, respectively. The mean distance between the ends of each chromosome, as well as the size of the nuclear diameter, appear indicated in **Table 2**. The ends of metacentric chromosome 3R and submetacentric chromosomes 4R and 5R, show very similar separation and the same happens in the group formed by metacentric chromosomes 2R and 7R and submetacentric chromosome 6R. There is no significant correlation between the inter-chromosome ends distance and the centromeric index (*r* = 0.471, *t* = 1.194, d.f = 5, and *p* > 0.05). Thus, chromosome conformation does not affect the telomere movement during the bouquet dissolution. Between chromosomes variation in the degree of separation of the two ends was correlated to the size of the nuclear diameter (*r* = 0.858, *t* = 3.73, d.f. = 5, *p* < 0.05). Such a variation should at least in part be a result of manipulation during the realization of preparations. In fact, with the exception of chromosome 3R, the between ends distance/nuclear diameter ratio was similar in all remaining chromosomes. The telomere dispersion produced in zygotene separated the chromosomal ends to nuclear positions distant on average 43% of the nuclear diameter.

**Table 2:** Mean separation between the two ends of each completely synapsed rye bivalent (S-L distance), average nuclear diameter (ND) and S-L distance/ND rate in PMCs at pachytene of wheat-rye additions.

Chromosome	S-L distance	ND	S-L distance/ND	PMCs
	(μm)	(μm)		
1R	21.6 ± 1.9	48.4 ± 1.6	0.45	143
2R	25.8 ± 2.7	61.8 ± 2.7	0.42	101
3R	19.4 ± 2.2	52.4 ± 2.3	0.37	107
4R	21.6 ± 2.3	50.9 ± 2.1	0.42	115
5R	18.8 ± 3.1	41.0 ± 2.9	0.46	43
6R	22.7 ± 2.3	48.4 ± 2.2	0.47	94
7R	26.0 ± 2.7	61.2 ± 2.4	0.42	126


### Rye Chromosome Arms Differ in the Level of Synapsis of Their Distal Half

**FIGURE 6:**
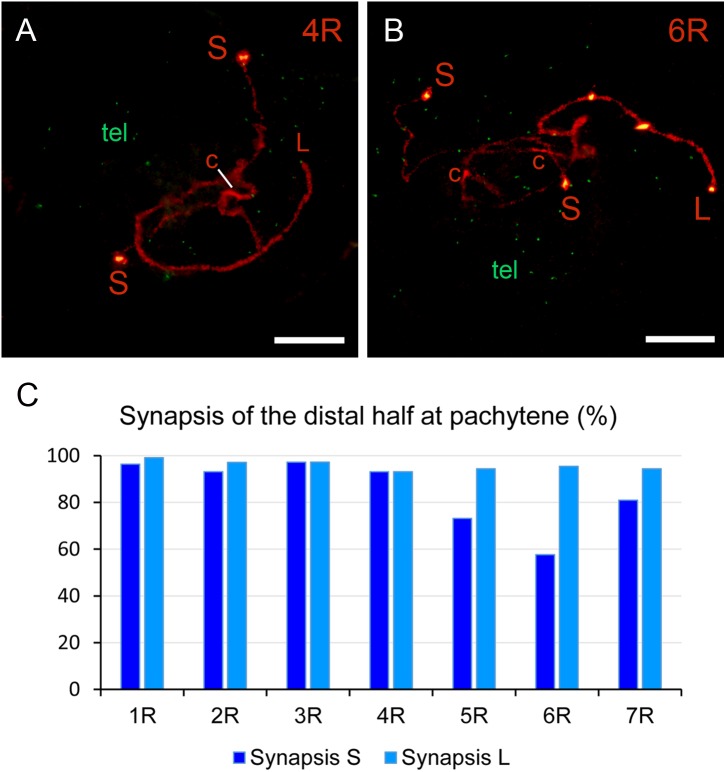
Synapsis of rye chromosomes in nuclei at pachytene in wheat-rye additions. **(A,B)** Nuclei showing partial synapsis of chromosomes 4R **(A)** and 6R **(B)**. The ends of the short (S) and long (L) arms, the rye centromeres (c) and all telomeres (green) are shown. **(C)** Level of synapsis in the distal half of both arms of the seven rye chromosomes. Bars represent 10 μm.

In many plant species crossovers are non-random distributed along the chromosomes. In wheat, barley and rye, they are confined to the distal half of each chromosome arm (Lukaszewski and Curtis, 1993; Akhunov et al., 2003; Lukaszewski, 2008; Valenzuela et al., 2012; Higgins et al., 2014). Such regions should undergo synapsis in order to facilitate the occurrence of crossovers. Most rye pachytene bivalents showed synapsed the distal half of both chromosome arms (**Figures 2C,E**, **5A,B**), however, unmatched chromosome arms were also observed (**Figures 6A,B**). Failure of synapsis in the distal half varied between chromosomes arms (**Figure 6C** and **Table 3**). Chromosome arms 5RS and 6RS, with disturbed telomere migration during leptotene, showed the lowest level of synapsis. Chromosome arm 4RS, despite restricted telomere migration, completed synapsis in most PMCs. Because synapsis progresses from the end to the center of the chromosomes (Corredor et al., 2007), unsynapsed stretches were more often observed in proximal regions, especially in chromosomes 6R and 7R (**Table 3**).

**Table 3:** Frequency (%) of PMCs with synapsis involving the distal half or the proximal half of individual rye chromosome arms at pachytene in disomic wheat-rye additions.

Chromosome	Distal half of the S arm	Proximal half of the S arm	Distal half of the L arm	Proximal half of the L arm	PMCs
1R	96.4	88.1	99.1	96.4	194
2R	93.1	82.2	97.1	86.1	101
3R	97.2	95.3	97.2	95.3	107
4R	93.1	87.0	93.1	84.7	131
5R	73.2	66.2	94.4	87.3	71
6R	57.7	57.7	95.4	78.5	130
7R	81.0	61.9	94.4	57.9	126


### Chiasma Formation Is Affected in Arms That Fail to Synapse and in Arms With a High Level of Synapsis

**FIGURE 7:**
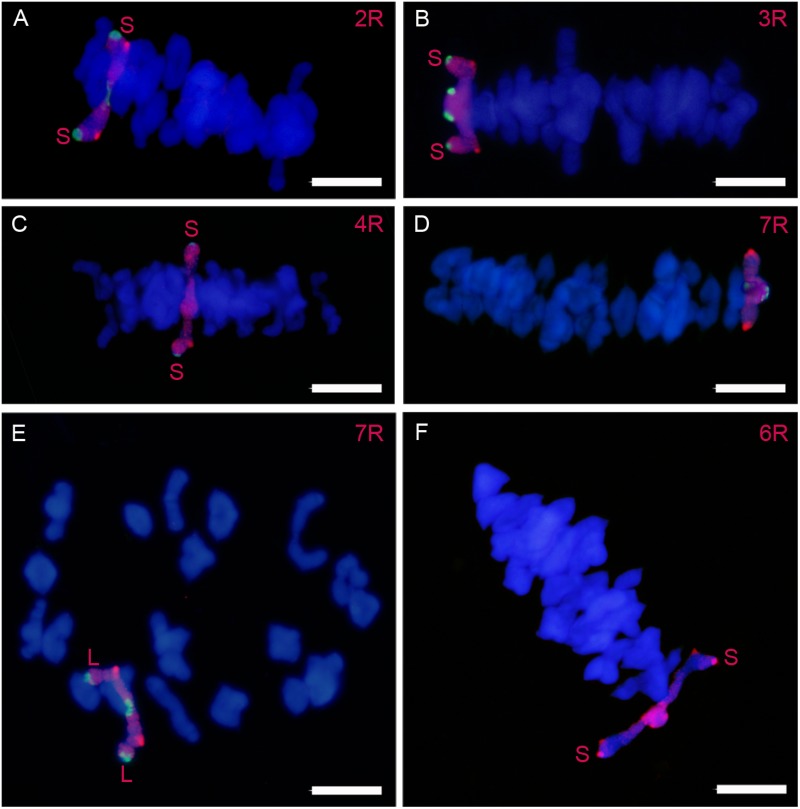
Rye bivalents in cells at metaphase I in wheat-rye additions. An open bivalent with the long arm bound is formed by chromosomes 2R **(A)**, 3R **(B)**, 4R **(C)**, and 6R **(F)** The long arm is bound in the open bivalent formed by chromosome 7R **(E)**. Chromosome pair 3R **(D)** forms a ring bivalent. Bars represent 10 μm.

Chiasmata formed by rye chromosomes were inferred from the occurrence of association between each homologous arm pair in PMCs at metaphase I (**Figure 7**). Two homologous arms bound at metaphase I had formed at least one chiasmata, but it is difficult to establish whether additional chiasmata were present or not. Thus, the frequency of homologous arms association at metaphase I represents an underestimation of the number of chiasmata per chromosome arm. **Table 4** shows the frequency of association for the different rye chromosome arms as well as the total number of associations per cell. Both arms of chromosomes 1R, 3R, and 7R, and the long arm of chromosome 2R formed chiasmata with frequencies higher than 90%. The remaining arms showed lower frequencies, especially the arms 2RS, 4RS, 4RL, 5RS, and 6RS, whose frequencies ranged between 25 and 70%. The total number of associations varied between lines. A maximum number of 42 arm associations can be formed in the wheat-1R line and 44 in the other six lines. Thus, chiasma formation failed not only between rye chromosome arms but also between wheat chromosomes, especially in the addition lines wheat-4R and wheat-5R.

**Table 4:** Frequency (%) of association of individual rye chromosomes arms and total number of bounds in cells at metaphase I of disomic wheat-rye additions.

Chromosome	S arm	L arm	Total bounds	PMCs
1R	91.1^a^	98.9^a^	41.7 ± 0.1	90
2R	67	96	41.3 ± 0.2	100
3R	95	96	43.3 ± 0.2	100
4R	46	70	37.9 ± 0.2	100
5R	36.4^a^	83.2^a^	39.7 ± 0.1	173
6R	25^a^	86^a^	40.9 ± 0.1	200
7R	92	98	42.4 ± 0.4	100


*^*a*^After Naranjo (2014).*

## Discussion

The majority of rye chromosomes of the addition lines studied undergo a meiotic behavior that it is different from that observed in rye itself. This is apparent, if attention is paid to the number of chiasmata formed. In rye, a total of 13.4 (1.9/chromosome) chiasmate bonds are formed at metaphase I (Naranjo and Lacadena, 1980). This figure represents an association frequency of 95.7% per chromosome arm. In the wheat-rye additions, the arms 2RS, 4RS, 4RL, 5RS, and 6RS show much lower frequencies of association (<71%, **Table 4**). But let us analyze separately the different steps of prophase I that precedes chiasma formation.

### Chromatin Remodeling

Remodeling of chromatin produced in leptotene leads to a more relaxed chromosome organization, which most likely facilitates the search of the homologous partner. During the SC assembly, the axial elements (chromosomes) become shortened. This shortening has been quantified in electron microscopic studies of spread nuclei of both rye (Gillies, 1985) and wheat (Martínez et al., 2001). The length of SCs at pachytene is approximately 80% of the length in zygotene. One can assume that rye chromosomes suffered a similar shortening in the course of zygotene in wheat-rye addition lines. According to the length of pachytene bivalents in the wheat-rye additions studied, rye chromosomes fall into two categories, one corresponds to chromosome 4R, with a length of 172.8 μm, and the other contains the six remaining chromosomes, with an average length of 100.6 μm. Such a divergence does not exist in rye itself. The length of SC bivalents at pachytene in two different stocks of rye, ranged between 72.2–61.8 μm and 64.0–54.1 μm, respectively (Gillies, 1985). Thus, the appearance of two different chromatin reorganization patterns between the chromosomes of rye suggests chromosome-specific responses to the new genetic background in which they have been introgressed.

One could assume that differences in length between chromosomes in pachytene might be a result of differences in their DNA content, given the large size, 8.1 Gb, of the rye genome (Doležel et al., 1998). Chromosome 4R consists of a DNA sequence of 1435 Mb, which represents 17.4% of the rye genome. This sequence is higher than those of the other six chromosomes, which range between 1023 Mb (1R) and 1253 Mb (2R) with an average size of 1136 Mb/chromosome (Martis et al., 2013). Taken into account the chromosomal length, the group of six chromosomes shows a mean packaging level of 11.3 Mb per μm in the pachytene bivalents while the ratio decreases to 8.3 Mb per μm in the case of 4R. The packaging level of chromosome 4R represents 73% of that observed in the other chromosomes. Thus, differences between chromosomes in their length at pachytene are the result, not only of differences in the DNA content, but also of distinct patterns of chromatin reorganization during early prophase I.

It is also noteworthy that the mean length of rye chromosomes at pachytene is much higher in the addition lines, 110.9 μm, than in rye itself, 62.5 μm (Gillies, 1985). The mean length of 110.9 μm found in the wheat-rye additions fits the average length of 90.2 μm of wheat chromosomes (Martínez et al., 2001). This variation of the mean chromosome length supports that the pattern of chromatin remodeling of a given chromosome at leptotene is controlled by the genetic background in which such a chromosome is present. In pachytene bivalents, the two homologues are closely juxtaposed along their entire length by the installation of the SC. Each chromatid of the bivalent consists of a linear arrangement of loops whose bases are anchored to the SC lateral element. Axes of sister chromatids are closely juxtaposed and their loops project out of the SC (Zickler and Kleckner, 2015). The density of loops along the pachytene axis is quite conserved in different organisms (∼20 loops per micron) (Kleckner, 2006). The evolutionarily conserved loop spacing implies that variation in the chromosome axis length should be accompanied by inversely correlated differences in the loops size. This is manifested in mice mutants that have altered the meiotic-specific cohesin Smc1β and the SC lateral component Sycp3; relatively long chromosome axes project short loops while shorter chromosome axes are coupled with longer loops (Revenkova and Jessberger, 2006; Novak et al., 2008). Thus, the increase of the length of rye pachytene bivalents in wheat-rye addition lines relative to plants of rye, as well as differences between lines, should be the result of modifications in the number and size of the loops.

Little is known about the impact of molecular interactions underlying the introgression of rye chromatin in a wheat background. A comparison of the transcriptomes of wheat, barley and a wheat-barley ditelosomic 7HL addition revealed reprograming of the transcriptomes of wheat and 7HL in the addition line (Rey et al., 2018). Only 3% of wheat genes underwent a different transcription rate in the addition line relative to wheat, while 42% of genes of 7HL were up- or down-regulated in the addition line relative to barley. These results evidence the existence of interactions between genes of both species, which may change with the introgressed chromosome. Thus, chromosome-specific molecular interactions affecting genetic functions related to the reorganization of chromatin during prophase I could cause differences between lines. Whether comparable genetic interactions exist or not in other sets of wheat-rye additions is unknown. This point may be addressed in future research works.

### Clustering and Dispersion of Telomeres

Telomeres of the long arms 1RL, 2RL, 3RL, 6RL, and 7RL as well as telomeres of the short arm of metacentric chromosomes 2R, 3R, and 7R, were included in the telomere cluster in almost all PMCs. By contrast, the telomere of the short arm of submetacentric chromosomes 4R, 5R, and 6R showed a lower probability of inclusion in the telomere cluster. This result reinforces previous studies supporting a chromosome conformation dependence in the ability of telomeres to converge in the telomere pole (Naranjo et al., 2010; Naranjo, 2014). Chromosome 1R with a centromeric index lower than that of 4R shows, however, a behavior more similar to that of metacentric chromosomes. It is possible that the presence in its short arm of the nucleolar organizing region, where chromatin is more relaxed, could affect the arrangement of this chromosome during leptotene.

The organization of the bouquet is the result of three interdependent events: the attachment of telomeres to the cytoskeleton through a nuclear envelope protein bridge, the oriented telomere cytoskeleton-mediated movement, and the clustering of telomeres in a small region of the nuclear membrane. Some of these steps were affected in the telomeres of the arms 4RS, 5RS, and 6RS. The link between the cytoskeleton and the chromosome ends during meiosis is provided by a complex of proteins among which are the transmembrane SUN (sad1/UNC-84) domain protein that interact with the chromosome ends (Starr, 2009) and the KASH (Klarsicht/Anc/Syne homology) domain protein that in turn interact with elements of the cytoskeleton (Starr and Fridolfsson, 2010). The function that the SUN and KASH proteins develop in fungi and animals is conserved in the plant kingdom from the most ancestral plant species to advanced angiosperms (Poulet et al., 2017). In maize and *Arabidobsis*, SUN protein foci spread through the nuclear envelop during leptotene, cluster in the periphery of one hemisphere at the bouquet, and back to spread through all the nuclear envelop during and beyond pachytene (Murphy et al., 2014; Varas et al., 2015). This SUN proteins distribution suggests that any chromosome end, regardless its location, has the possibility of finding its attachment to the nuclear envelope prior to migration. Thus, failure of the attachment of telomeres of 4RS, 5RS, and 6RS to the nuclear envelope does not seem the reason of their disturbed positioning.

Another explanation of the behavior of telomeres of 4RS, 5RS, and 6RS is that they have to cover longer distances than other telomeres to reach the telomere pole during the bouquet organization. This explanation is based under the assumption that the site of the telomere pole is most likely installed in the region of the telomeric hemisphere with the highest density of telomeres, that is, in the region where telomeres of long arms are located. In such a situation, telomeres of metacentric chromosomes can be equidistant relative to the telomere pole, while telomeres of the short arms of submetacentric chromosomes lay farther away of this pole. In support of this assumption are two facts: (i) telomeres of the long arms are included in the telomere cluster in almost all cells, (ii) the centromeric end of telocentric chromosomes fails also in its incorporation to the telomere pole (Corredor and Naranjo, 2007). The telomere of the centromeric end of the 5RL telocentric locates at the centromere pole at early leptotene. In the course of leptotene this chromosome end abandons the centromere pole to cluster with the other telomeres in the opposite pole. However, only 51% of the centromeric ends incorporate into the telomere cluster at the consolidated bouquet stage.

The effect of chromosome conformation on telomere clustering is not extended to the movement produced during zygotene, which disperses telomeres through all the nuclear periphery. Telomeres of any chromosome pair are very close at the bouquet and their separation seems to be at random, i.e., without any programed itinerary. Within lines variation of the distance between the ends of the rye bivalent at pachytene agrees with a random dispersion of telomeres. Most likely, the overall arrangement of bivalents in the nucleus and the simultaneous movement of all chromosomes should condition severely the distance covered by each telomere.

### Synapsis and Chiasma Formation

Restriction of chiasma formation to the distal half of chromosome arms in species such as wheat, rye or barley (Lukaszewski and Curtis, 1993; Akhunov et al., 2003; Lukaszewski, 2008; Valenzuela et al., 2012; Higgins et al., 2014) implies that such chromosome regions should develop synapse regularly. This was so for most rye chromosome arms. All of the arms with normal telomere migration during the bouquet organization showed normal synapsis. Failure of synapsis affected mainly the short arm of submetacentric chromosomes 5R and 6R. Synapsis failure in these arms was preceded of disturbed migration of their telomere during the bouquet formation, which most likely impeded the occurrence of physical interactions and synapsis initiation between their subtelomeric homologous. The behavior of 5RS and 6RS is in contrast with that of 4RS, which completed synapsis despite its telomere failed also to be included in the telomere cluster. What makes chromosome arm 4RS different? This arm reaches a much higher length at pachytene than 5RS and 6RS. Unfolding of chromatin produced in leptotene obliges chromosomes to move and span the entire nucleus because their length exceeds largely the nuclear diameter. The intensity of the movement is expected to increase with the length reached by each chromosome. Such a chromosome dynamics might promote the occurrence of chance encounters between intercalary homologous regions regardless their respective telomeres were separated. Under this assumption, the probability of intercalary interactions between homologues is expected to be higher in chromosome arm 4RS than in 5RS and 6RS. Synapsis initiated intercalarily in 4RS could extend later to its distal region, although the telomeres were not initially associated. This situation is comparable to that found in an inversion heterozygote for the long arm of chromosome 1R. In this heterozygote, the crossover-rich region is positioned distally in the standard chromosome and proximally in the inverted chromosome at the initiation of meiosis, i.e., they are located in opposite poles of the nucleus. However, during zygotene, a non-telomere led chromosome movement, the movement generated by chromosome elongation, makes it possible that these homologous regions find one another and that the entire arm complete synapsis (Lukaszewski, 2008; Valenzuela et al., 2012; Naranjo, 2014). Thus, the high level of synapsis of chromosome 4R can be the result of homologous interactions facilitated either by the bouquet organization or, in cells with disturbed migration of the 4RS telomere, by the chromosome movement derived of chromatin unfolding. The distal half of the short arm of metacentric chromosome 7R showed a level of synapsis of 81%, which is higher than that of 5RS (73%) or 6RS (57%) but lower than the level of the remaining arms (**Table 2**). However, the frequency 92% of association of chromosome arm 7RS at metaphase I suggests an almost normal level of synapsis in the anther where metaphase I was studied.

Chiasma distribution in rye chromosomes of wheat-rye addition lines was earlier studied (Drögenmüller and Lelley, 1984). However, the number of chiasmate bonds per bivalent instead the association frequency of each chromosome arm was reported. Nevertheless, chromosomes 5R and 6R formed a more reduced number of chiasmata than the remaining rye chromosomes. Reduction of the number of chiasmata in chromosomes 5R and 6R affects mainly their short arm (**Table 4**) and is the consequence of the synapsis failure that such arms showed at pachytene. However, other arms with normal synapsis suffered a considerable reduction of the chiasma frequency. This was the case of the arms 2RS, 4RS, and 4RL. The wheat-4R addition line showed the lowest number of chiasmate bonds, 37.9, which is far from 44, the expected maximum number. Environmental variation could explain minor differences between lines, but the low chiasma frequency observed in the wheat-4R addition suggests a negative effect of the genetic background of this line on chiasma formation, which affects both wheat and rye chromosomes. Addition of individual chromosomes of rye to wheat produce changes in traits such as spike and spikelet morphology (Driscoll and Sears, 1971). Addition of chromosome 4R increases also the length of the anthers and the number of pollen grains, while the addition of 1R or 2R reduce fertility (Nguyen et al., 2015). It is possible that chromosome 4R carries some genetic information that interferes on chiasma formation. The wheat-2R addition forms, however, a high number of chiasmata, 41.3. The reduction observed in chromosome arm 2RS seems to be chromosome-specific and derived from the genetic constitution or chromatin organization of this arm. In fact, chromosome arm 2RS carries a large subtelomeric heterochromatin chromomere and such a heterochromatin has been shown to reduce the number of chiasmata of rye chromosomes in wheat-rye derivatives (Naranjo and Lacadena, 1980).

## Author Contributions

TN designed and conducted the research and wrote the work.

## Conflict of Interest Statement

The author declares that the research was conducted in the absence of any commercial or financial relationships that could be construed as a potential conflict of interest.
